# Overweight condition and waist circumference and a candidate gene within the 12q24 locus

**DOI:** 10.1186/1475-2840-12-2

**Published:** 2013-01-03

**Authors:** Claudia Gragnoli

**Affiliations:** 1 Laboratory of Molecular Genetics of Complex and Monogenic Disorders, Department of Medicine and Cellular & Molecular Physiology and Biostatistics, M. S. Hershey Medical Center, Penn State University College of Medicine, Hershey, PA, USA; 2 Center for Biotechnology and Department of Biology, Temple University’s College of Science & Technology, Philadelphia, PA, USA; 3 Molecular Biology Laboratory, Bios Biotech Multi-Diagnostic Health Center, Rome, Italy

**Keywords:** *PSMD9*, SNP, Linkage, 12q24, Overweight, Waist

## Abstract

**Aims:**

Obesity and obesity-associated phenotypes are linked to the chromosome12q24 locus, the non-insulin-dependent-diabetes 2 (NIDDM2) locus. The gene of proteasome modulator 9 (*PSMD9)* lies in the NIDDM2 region and is linked to type 2 diabetes (T2D), microvascular and macrovascular complications of T2D.

We aimed at studying whether the *PSMD9* T2D risk single nucleotide polymorphisms (SNPs) *IVS3+nt460*, *IVS3+nt437*, and *197G* are linked to obesity, overweight status and waist circumference in Italian T2D families.

**Methods and results:**

We screened 200 Italians T2D siblings/families for PSMD9 variants. Using Merlin software, we performed non-parametric linkage analysis to test for linkage with obesity and overweight condition and variance component analysis to test for linkage with waist circumference in our T2D siblings/families dataset.

Our study shows that the *PSMD9* SNPs *IVS3+nt460*, *IVS3+nt437*, and *197G* are in linkage with overweight condition and waist circumference in Italians. The statistical power tests performed via simulations on real data confirm that the results are not due to random chance.

**Conclusions:**

In summary, the linkage strategy using a homogeneous family/subject dataset can identify a gene contributing to a complex trait. *PMSD9* may be at least one of the genes responsible for the linkage to obesity and obesity-associated phenotypes at the locus 12q24 in other populations.

## Introduction

Several groups reported linkage within the NIDDM2 (non-insulin-dependent diabetes mellitus) locus at chromosome 12q24 to T2D, obesity, central fat mass and waist circumference [1-8]. Within the 12q24 locus lies the gene of proteasome modulator 9 (*PSMD9)*, a transcriptional regulator of the insulin gene, highly expressed in pancreatic islets [9]. PSMD9 is a ubiquitous protein of eukaryotic cells and part of the 26S proteasome complex, contributing to intracellular proteins degradation into antigenic peptides in the immune response to antigen presentation by MHC class I cells (http://www.genecards.org/cgi-bin/carddisp.pl?gene=PSMD9). PSMD9 may play a role in inflammation as part of the autoimmune process [10]. Further, PSMD9 regulates the transcription of the ligand-dependent retinoid-target genes (http://www.millipore.com/pathways/pathviewer.do?pathwayId=76). Since PSMD9 is a coactivator of insulin gene transcription [9], and in pancreatic overexpression of transgenic mice cause diabetes [11], *PSMD9* variants may contribute to T2D as well as to obesity, overweight status and visceral obesity.

We reported that *PSMD9* may rarely cause T2D by unique mutations [12] and that is linked to T2D [13], maturity-onset diabetes of the young 3 (MODY3) [14], T2D-microvascular [15-18] and T2D-macrovascular complications [19], hypercholesterolemia [20], hypertension [21], carpal tunnel syndrome [22], and depression [23]. Of note, the 12q24 locus is not only linked to T2D [24], macrovascular [25,26] and microvascular disease [27], but also to dyslipidemia [28], hypertension, obesity, [2], body mass index and C-reactive protein jointly [29], cat-specific IgE and total IgE in asthma [30], and most interestingly to bipolar disorder [31] and depression [32]. Also a recent study has reported that a deletion at the locus 12q24.33 is significantly correlated with height, suggesting that this locus is important in determining height and may contribute to height variation in human populations [33].

The aim of the present study is to identify whether *PSMD9 IVS3+nt460*, *IVS3+nt437*, and *197G* single nucleotide polymorphisms (SNPs) may be linked to maximum lifetime obesity, maximum lifetime overweight status and visceral obesity measured by waist circumference in an Italian family dataset.

## Methods

### Ethics statement

The subjects were all recruited from center Italy following the Helsinki declaration guidelines. Subjects gave written informed consent and the Penn State College of Medicine Ethical Committee approved the study.

### Families

We recruited 200 Italian T2D affected siblings and families. We excluded all families with identical twins and confirmed that at least three generations were Italian. Italians are a homogeneous population living in a peninsula and they are suitable for genetic studies due to lack of genetic admixture. This family dataset has helped studying and identifying T2D and MODY genes contributing to disease [34-48].

### Clinical phenotyping

We characterized the Italian families for maximum lifetime body mass index [BMI=weight in Kg/(height in m)^2^], indicating as overweight those with maximum lifetime BMI ≥25 and as obese those with maximum lifetime BMI ≥30. We measured also the waist circumference in cm. BMI and waist circumference during pregnancy in females were not considered. Phenotypes are described as unknown if diagnosis is unclear because data are lacking. In our 200 Italian families, data are less than 100% for each phenotype.

### Sequencing

Via PCR, we amplified with specific primers in the affected and unaffected family members the *IVS3 PSMD9* region containing the *+nt460 A/G* and *+nt437 C/T* SNPs and the exon 5 coding region containing the *E197G A/G* SNP. We directly sequenced the PCR products, status post-purification via EXOSAP-IT on an automated ABI 3730 Sequencer.

### Linkage analysis

We tested in the 200 Italians families for linkage of the *PSMD9* SNPs with maximum lifetime obesity, maximum lifetime overweight condition and waist circumference.

The non-parametric linkage analysis for the qualitative phenotypes and the variance component analysis for the quantitative trait were performed by using Merlin software [49], calculating the allelic frequency from the total 200 genotyped families. Merlin analyzed all the informative families within this dataset. All results are reported as LOD scores calculated by Merlin.

### Simulations

For each test performed, to exclude the presence of any results due to random chance, we calculated how many times similar P-values were expected by chance in 1,000 replicates of simulations by using the gene dropping method: this analysis replaces real data with simulated data, while maintaining the pedigree structure, allele frequencies and recombination fraction. These datasets are generated under the null hypothesis of no linkage.

## Results

The results (LOD score, P-value and empirical P-value) of the non-parametric linkage analysis performed for the qualitative phenotypes overweight status and obesity are shown in Table 1.

**Table 1:** Non-parametric linkage analysis of phenotypes of the 200 Italian families by Merlin software

**Phenotype**	**Prevalence**	**Families**	**Lod score**	**P-value**	**Empirical P-value**
**Overweight**	85.20%	112	1.22	0.009	0.004
**Obesity**	47.50%	42	0.62	0.050	0.053

Prevalence = phenotype prevalence among the family subjects studied; Families = families number analyzed; Lod score = derived from the non-parametric linkage analysis by Merlin; Empirical P-value = derived from 1000 replicates by using the gene dropping method; Overweight = presence of BMI ≥25; Obesity = presence of BMI ≥30.

The results of the variance component analysis performed for the quantitative trait waist circumference in cm with the LOD score, P-value, empirical P-value, trait heritability and trait heritability attributable to the SNPs under study are reported in Table 2*.*

**Table 2:** Variance component linkage analysis of the waist circumference in cm of the 200 Italian families by Merlin software

**Quantitative trait**	**Families**	**Trait heritability**	**Gene trait heritability**	**Lod score**	**P-value**	**Empirical P-value**
**Waist in cm**	108	50.66%	49.17%	0.93	0.020	0.023

Families = families number analyzed; Lod score = derived from the quantitative trait analysis by Merlin; Empirical P-value = derived from 1000 replicates by using the gene dropping method; Trait Heritability = Heritability of the quantitative trait calculated by Merlin; Trait Heritability attributable to the *PSMD9* SNPs.

## Discussion

Our analyses show that the *PSMD9 IVS3+nt460*, *IVS3+nt437*, and *197G* SNPs are in linkage with maximum lifetime overweight and with waist circumference, representing visceral obesity. Further, there is a trend towards linkage with obesity. The reduced number of families meeting the criteria for obesity may have reduced the power to detect linkage.

The 12q24.2 chromosome bears the NIDDM2 locus, the MODY3 gene *HNF-1α*, cause of T2D and MODY3, respectively. We previously reported that unique rare *PSMD9* mutations contribute to T2D and that the *IVS3+nt460*, *IVS3+nt437*, and *197G* SNPs are linked to T2D and MODY3 in our Italian dataset [12-14]. Of note, *PSMD9* linkage to micro- and macro-vascular disease of T2D [15-19], hypercolesterolemia [20], hypertension [21] and depression [23] suggest a potential role in mediating an inflammatory response, which could explain the presence of linkage to the obesity-associated phenotypes.

Our results demonstrating the presence of linkage to overweight condition as well as to waist circumference are encouraging as the same locus is in linkage in other populations with obesity, central fat mass and waist circumference [1,2,8]. The data should be replicable in other ethnic groups and are of high interest to the groups who revealed linkage in the same locus, which may be explained by the *PSMD9* variants.

In addition, we again prove the strength and validity of the linkage approach in identifying genes in complex disorders. In fact, family-based linkage studies are safer in their statistical nature compared to association studies. Also, we consider that the use of microsatellites as markers in the past may have negatively affected the results of the linkage, especially in polygenic diseases and with the use of affected siblings, as these markers may undergo duplications and mutations in vivo. In fact, previously we could not identify linkage with T2D at 12q24 using multiple highly informative and polymorphic microsatellites within the same family dataset (data not shown) in which the linkage was identified using SNPs [13]. Hence, SNP-based linkage studies are more reliable. Also, the family dataset should be ethnically homogenous to allow results to be revealed.

*PSMD9* should be screened in other populations for mutations and common variants in families with obesity, overweight condition and visceral obesity.

Interestingly, the reported 12q24 locus linkage to cat-specific IgE and total IgE in asthma [30] could offer a possible explanation of the pleiotropic effects of such locus. In fact, the identified 12q24 linkage to multiple disorders including T2D [24], micro- and macrovascular disease [25-27], hypertension [2], body mass index and C-reactive protein [29], dyslipidemia [28], depression [32] and bipolar disorder [31] supports the idea that there could be a common underlying pathogenetic factor triggering the above-mentioned multiple disorders, such as an exaggerated inflammatory response or a deregulated autoimmune response leading to disease status. And this hypothesis fits well with the role of PSMD9 in the proteasome complex 26S processing the antigens for the immune response (http://www.genecards.org/cgi-bin/carddisp.pl?gene=PSMD9). PSMD9 may play an inflammatory role in autoimmunity [10] with pleiotropic effects. Of note, the finding that 12q24.33 deletion affects height [33] could represent an outcome of the inheritance of factors predisposing to pre-disease states and thus affecting adult final growth. In fact, height is inversely associated with cardiovascular disease [50] and cardiovascular risk factors, such as insulin resistance [51]. Thus, the 12q24 locus could bear the gene or genes contributing to waist circumference, insulin resistance, cardiovascular disease, depression and height determination. It would be of interest to study the role of PSMD9 in asthma and IgE-mediated disorders as well as in final adult height.

## Competing interest

The author has not conflict of interest to declare.
